# The penicillin binding protein 1A of *Helicobacter pylori*, its amoxicillin binding site and access routes

**DOI:** 10.1186/s13099-021-00438-0

**Published:** 2021-06-28

**Authors:** Bahareh Attaran, Najmeh Salehi, Bahareh Ghadiri, Maryam Esmaeili, Shadi Kalateh, Mohammad Tashakoripour, Mahmoud Eshagh Hosseini, Marjan Mohammadi

**Affiliations:** 1 HPGC Research Group, Department of Medical Biotechnology, Pasteur Institute of Iran, Tehran, Iran; 2 Department of Microbiology, Faculty of Biological Sciences, Alzahra University, Tehran, Iran; 3 Department of Bioinformatics, Institute of Biochemistry and Biophysics, University of Tehran, Tehran, Iran; 4 Gastroenterology Department, Amiralam Hospital, Tehran University of Medical Sciences, Tehran, Iran

**Keywords:** *H. pylori*, Amoxicillin, Resistant, PBP1A, S414R, V469M, Thr556Ser, Binding site, Access tunnel

## Abstract

**Background:**

Amoxicillin-resistant *H. pylori* strains are increasing worldwide. To explore the potential resistance mechanisms involved, the 3D structure modeling and access tunnel prediction for penicillin-binding proteins (PBP1A) was performed, based on the *Streptococcus pneumoniae*, PBP 3D structure. Molecular covalent docking was used to determine the interactions between amoxicillin (AMX) and PBP1A.

**Results:**

The AMX-Ser368 covalent complex interacts with the binding site residues (Gly367, Ala369, ILE370, Lys371, Tyr416, Ser433, Thr541, Thr556, Gly557, Thr558, and Asn560) of PBP1A, non-covalently. Six tunnel-like structures, accessing the PBP1A binding site, were characterized, using the CAVER algorithm. Tunnel-1 was the ultimate access route, leading to the drug catalytic binding residue (Ser368). This tunnel comprises of eighteen amino acid residues, 8 of which are shared with the drug binding site. Subsequently, to screen the presence of PBP1A mutations, in the binding site and tunnel residues, in our clinical strains, in vitro assays were performed. *H. pylori* strains, isolated under gastroscopy, underwent AMX susceptibility testing by E-test. Of the 100 clinical strains tested, 4 were AMX-resistant. The transpeptidase domain of the *pbp1a* gene of these resistant, plus 10 randomly selected AMX-susceptible strains, were amplified and sequenced. Of the amino acids lining the tunnel-1 and binding site residues, three (Ser414Arg, Val469Met and Thr556Ser) substitutions, were detected in 2 of the 4 resistant and none of the sequenced susceptible strains, respectively.

**Conclusions:**

We hypothesize that mutations in amino acid residues lining the binding site and/or tunnel-1, resulting in conformational/spatial changes, may block drug binding to PBP1A and cause AMX resistance.

**Supplementary Information:**

The online version contains supplementary material available at 10.1186/s13099-021-00438-0.

## Background

*Helicobacter pylori* is a prevalent etiologic agent for chronic gastritis, gastric and duodenal ulcers, and in rare cases, gastric adenocarcinoma [1]. A global systematic review concluded that approximately 4.4 billion individuals are positive for *H. pylori* infection worldwide, and its prevalence varies from 18.9 to 87.7% of the populations [2]. This infection is also associated with an increased incidence of extra-gastric diseases, such as cardiovascular, respiratory, hepatic, and allergic diseases [3]. Successful eradication of *H. pylori* infection would effectively reduce the prevalence of the mentioned complications, especially gastric cancer, and is therefore considered as one of the controllable factors in the process of gastric carcinogenesis [4].

Amoxicillin (AMX), as a bacterial cell wall synthesis inhibitor, is a common constituent of first-line and rescue treatment, due to its high efficiency and fewer side effects [5]. Its use is recommended in a 14-day quadruple treatment regimen and 10-day sequential treatment [6]. A recent meta-analysis, comprising 66,142 clinical isolates from 178 studies, of 65 countries, declared up to 10 percent primary resistance to AMX in clinical *H. pylori* strains [7].

AMX belongs to the beta-lactam family of antibiotics that binds the penicillin-binding proteins (PBPs) [8]. Bacterial PBPs are membrane-associated enzymes, whose activities are essential for cell division and are classified into low-molecular-mass (LMM) and high-molecular-mass (HMM) categories [9, 10]. PBPs are responsible for glycosyltransferase and transpeptidase activities that lead to cross-linking of d-alanine and d-aspartic acid in bacterial cell walls [11]. Crosslinking adjacent peptidoglycan strands, via peptide stems, is essential for bacterial cell wall integrity and cell viability [11, 12]. HMM-PBPs constitute the main targets of β-lactam antibiotics, including AMX [13, 14]. Bacterial resistance to AMX is mainly due to the production of β-lactamase or structural alterations in one of the PBPs, involved in cell wall synthesis.

*Helicobacter pylori* seem to differ in this regard, as it is evidenced that point mutations in the *pbp1a* gene are the main reason for its AMX-resistance [15, 16]. Nine different PBPs have been reported for *H. pylori*; 3 HMM, including PBP1 (72 kDa), PBP2 (62 kDa) and PBP3 (54 kDa) [17, 18], and 6 LMM (PBP4-9) with 50, 44, 35.5, 33, 28 and 21 kDa molecular weights, respectively [17, 19, 20]. Class A PBPs have both glycosyltransferase and transpeptidase activities, whereas class B PBPs possess only the latter. Furthermore, the combination of these two enzymatic activities of PBP1A is essential for cell wall homeostasis [21]. AMX has binding affinities for PBP1, PBP2, and PBP3. However, in resistant *H. pylori* strains, its affinity for PBP1A is significantly diminished [18]. Accordingly, mutations in PBP1A are considered the predominant cause of AMX resistance in *H. pylori* [15, 22, 23].

Using homology modeling, the role of previously reported amino acid substitutions of *H. pylori* PBP1A, in binding to AMX has been carefully analyzed [24]. However, no crystal structure information is available on the *H. pylori* PBP1A or its PBPs in general. Consequently, the exact locations of the active and antibiotic binding sites remain to be explored. In this study, we carried out covalent docking analysis of PBP1A with AMX, to characterize the interactions between AMX and its binding site, as well as to identify the potential drug access routes. Subsequently, we evaluated any existing mutations of these residues, in our few resistant clinical strains of *H. pylori,* in correlation with their drug susceptibility.

## Results

### Structure prediction of PBP1A and covalent molecular docking with AMX

**Fig. 1:**
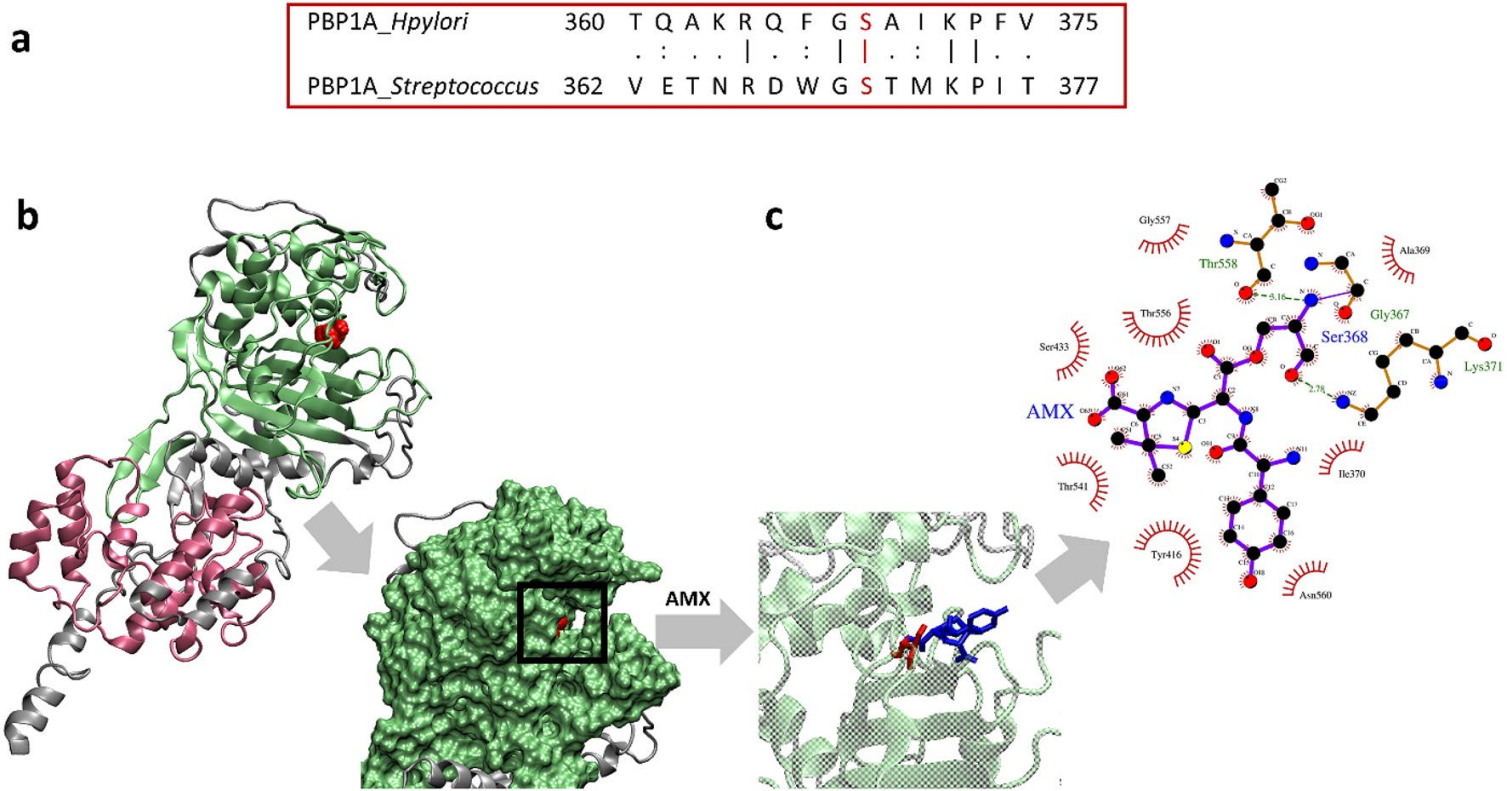
The PBP1A sequence, structure in *H. pylori* and its interaction with AMX. **a** A segment of the pairwise sequence alignment of PBP1A in *H. pylori* and *Streptococcus.* The equivalent Ser368 and Ser370 in *H. pylori* and *Streptococcus* are shown in red. **b** The 3D structural model, transglycosylase, and transpeptidase domains are shown in silver, pink, and green cartoon secondary structures, respectively. The surface of the transpeptidase domain and the binding site are depicted in the inset figure. **c** The formation of the covalent interaction between the AMX and Ser368. The Ser368 and AMX are colored in red and blue, respectively. In the 2D panel, the non-covalent interaction between AMX-Ser368 and other PBP1A residues are presented. The protein residues involved in hydrophobic contacts and hydrogen bonds are represented in red spoked arcs and green dotted lines, respectively

The best 3D structural model of *H. pylori* PBP1A was built with the I-TASSER server, using the top 10 threading templates, shown in Additional file 1: File S1. This best model revealed the closest structural similarity to *Staphylococcus aureus* PBP2 (PDB ID: 3DWK) with 24.5% sequence identity, 87.4% sequence coverage, and an RMSD of 0.78 Å. The minimized 3D structure model of PBP1A is shown in Fig. 1b. MolProbity analysis on the Ramachandran plot of the model identified 86.23% of the residues to be in the favored regions, and only 4.23% stand as outliers (Additional file 2: Figure S1). The MolProbity score, which is on the same scale as the X-ray resolution and combines the clashscore, rotamer, and Ramachandran evaluations, was 1.76 for this structure. These results indicate that the minimized model has a reasonable quality for subsequent analysis. After model minimization and validation, covalent docking with AMX was performed.

X-ray crystallography of the antibiotic recognition site of PBP1A in *Streptococcus pneumonia*, has identified Ser370, as the catalytic residue that can form a covalent interaction with the β-lactams [25]. According to pairwise sequence alignment of PBP1A of *H pylori* and *Streptococcus pneumonia*, this residue is the equivalent of Ser368 in *H. pylori* (Fig. 1a). The insert in Fig. 1b shows the binding site of *H. pylori* PBP1A, which is relatively narrow. In the interaction of PBPs with β-lactams, the catalytic serine attacks the β-lactam ring and causes an acyl-enzyme complex [26]. To further explore this interaction, we have carried out the covalent docking of AMX with Ser368 of PBP1A. The Ser368 and AMX, which connect covalently, are shown in red and blue in Fig. 1c. As depicted in this figure, the AMX-Ser368 covalent complex interacts with Gly367, Ala369, ILE370, Lys371, Tyr416, Ser433, Thr541, Thr556, Gly557, Thr558, and Asn560, in the binding site of PBP1A, non-covalently. These residues were in agreement with the most probable binding residues of PBP1A, which were predicted by COACH (Additional file 3: File S2).

### The access routes to the AMX binding site in PBP1A

**Fig. 2:**
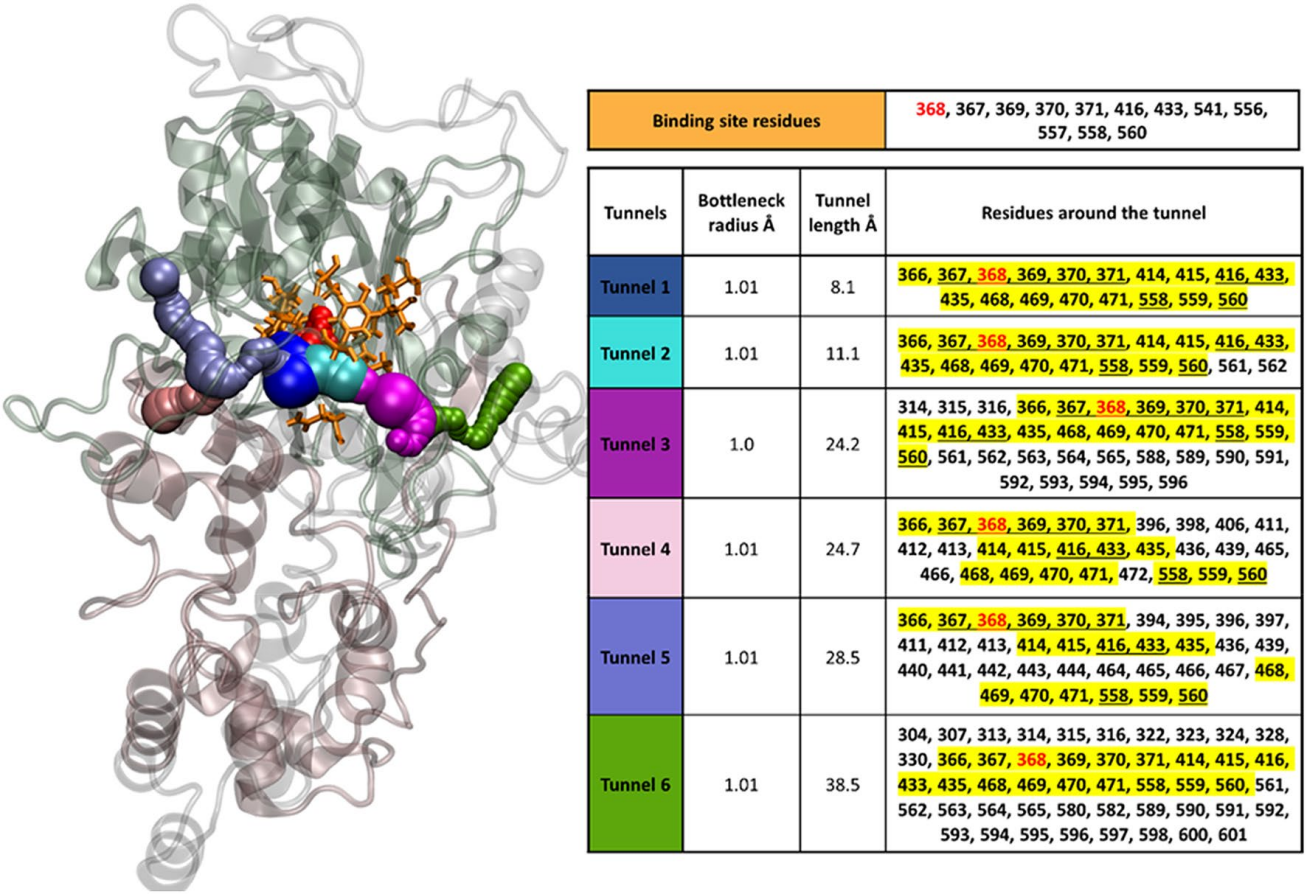
Presentation of the AMX binding site and access tunnels in PBP1A. The binding site residues and the six access tunnels are shown in orange, blue, cyan, magenta, pink, purple, green. Inset table: Amino acid residues of the binding site and those lining the access routes to the PBP1A binding site in *H. pylori* are listed. The tunnel cells are colored based on the tunnels in the 3D structure. The Ser368, which is connected covalently to the AMX, is depicted in red. The residues, commonly present in all tunnels are depicted in yellow highlights. The common residues of the binding site and tunnel-1 are underlined

As mentioned above, the binding site of PBP1A is very narrow, so any modifications to the binding site and/or its access routes may affect drug access. Using the CAVER tool, the potential access tunnels for PBP1A of *H pylori* were predicted (Fig. 2). These results showed six possible access routes for the ligand (AMX) to access the binding site. All six tunnels were identical in width (radius of ~ 1 Å), with varying lengths of 8.1 Å, 11.1 Å, 24.2 Å, 24.7 Å, 28.5 Å, and 38.5 Å, respectively. The amino acid residues lining the binding site and the six access tunnels are depicted in Fig. 2 and its inset table, respectively. Tunnel-1 is the final access route, leading to the drug binding site and its catalytic (Ser368) residue. This tunnel is comprised of 18 amino acid residues at the following positions: 366, 367, 368, 369, 370, 371, 414, 415, 416, 433, 435, 468, 469, 470, 471, 558, 559, and 560, which include the catalytic residue, as well as 8 residues of the drug binding site (inset Table of Fig. 2, underlined). The other five tunnels converge with tunnel-1 before reaching the drug binding site.

### The amoxicillin resistance rate and PBP1A mutations

We then evaluated mutations pertaining to the above listed residues, in our clinical *H. pylori* strains*,* in accordance with their drug susceptibility. Of the 100 clinical strains of *H. pylori* tested for amoxicillin susceptibility via E-test, 4 were found AMX-resistant. The transpeptidase domain of the *pbp1a* gene, which is considered its hypermutable region in *H. pylori*, was amplified and sequenced in these 4 AMX-resistant and 10 randomly selected AMX-susceptible strains. The sequences were aligned against the reference (ATCC: 26695 and J99) strains, and the detected amino acid substitutions in the binding site and tunnel-1 residues are depicted in Table 1 and Additional file 4: Figure S2. Of the above-listed residues, the only amino acid substitutions, namely Ser414Arg, Val469Met, and Thr556Ser, belonging to tunnel-1 or the binding site residues, were detected in 2 of the 4 AMX-resistant strains. Whereas, none of the listed residues were altered (mutated) in the 10 randomly sequenced sensitive strains (Table 1).

**Table 1:** Detected mutations in the PBP1A drug binding site and tunnel-1 residues of AMX- resistant and susceptible strains

Strains	Binding site	Tunnel-1	MIC (mg/L)
Ref			
J99	–	–	S
26,695	–	–	S
Sensitive			
MK984227	–	–	0.064
MK984220	–	–	0.064
MK984215	–	–	0.094
MK984213	–	–	0.125
MK984219	–	–	0.125
MK984223	–	–	0.125
MK984221	–	–	0.125
MK984226	–	–	0.094
MK984225	–	–	0.032
MK984214	–	–	0.125
Resistant			
MK984217	–	–	0.38
MK984216	–	Ser414Arg Val469Met	0.5
MK984218	Thr556Ser	Ser414Arg	0.38
MK984224	–	–	0.75

## Discussion

In recent decades, *Helicobacter pylori* resistance to antibiotics has significantly increased, thereby decreasing its eradication rate worldwide [27]. AMX, a β-lactam antibiotic, has long been a common constituent of first-line multiple drug therapy against *H. pylori* infection. The worldwide rate of AMX resistance was reported as an average of 4.55%, in a recent systematic review [27]. In accordance with the worldwide average rate, a 4 percent rate of resistance was detected in our study.

AMX-resistance causing factors include mutations in PBPs [28], β-lactamases [29], efflux pumps [30], and biofilm formation [31]. Point mutations in the *pbp1a* gene are considered as the leading cause of AMX resistance in *H. pylori* [16]. β-lactamases, although involved in AMX-resistance in other gram-negative bacteria, seem less critical in *H. pylori* [29, 32]. On the other hand, although mutations in *pbp2* and *pbp3* genes may also cause AMX resistance [28], those corresponding to the C-terminus of PBP1A protein, are considered as the main determinants of stable resistance in *H. pylori* [18]. The potential resistance provided by the PBP2X and PBP2B mosaics is limited by the presence of a “virgin” PBP1A, which still justifies particular effectiveness for β-lactam treatment. Thus, high level of resistance is dependent on an altered PBP1A [26].

In order to better understand this phenomenon, we used computational tools to analyze the interactions between AMX and PBP1A. In *Staphylococcus aureus*, PBPs form a stable covalent bond between their catalytic Ser370 residues and AMX, thereby preventing bacterial cell wall synthesis by inactivating the transpeptidase domain [33]. It is known that modification of amino acid residues lining the drug access tunnels affects the enzyme’s activity, specificity, enantioselectivity, and stability [34, 35]. In case of enzymes, such as xylanase, with buried binding sites, transporting substrates between active sites and the surrounding solution, through the access tunnels is a critical step in the catalytic cycle of these enzymes. Therefore, tunnel modification impacts the catalytic properties of enzymes [36]. It has been suggested that Lys371, Ser433, and Lys555 in *H. pylori* PBP1A, can form hydrogen bond interactions, with the putative catalytic Ser368 [24]. Our study has identified the common presence of Lys371 and Ser 433 amongst the binding site and tunnel-1 residues, and Gly367, Lys371, and Thr558 in hydrogen bond interaction with Ser368. Thr556 is another binding site residue, introduced as an important residue, in or adjacent to the penicillin-binding motifs [24]. Val469, one of the tunnel-1 amino acid residues, is also identified as one of the key residues in amoxicillin resistance, that is located in a loop enclosing the PBP1A binding site [24].

Then, to confirm our results, we evaluated mutations in the binding site and tunnel-1 residues, in our clinical *H. pylori* strains isolated under gastroscopy, which underwent AMX susceptibility testing. In addition, we performed a literature survey on the subject (Table 2). The our experimental data on our very limited number of resistant strains, identified Ser414Arg, Val469Met, and Thr556Ser substitutions (belonging to tunnel-1 and the binding site residues), in 2 of the 4 AMX-resistant and none of the 10 randomly sequenced sensitive strains. Accordingly, amino acid substitutions of binding site residues, including Ala369Thr (3 out of 4) [28] and Thr541Ile (1 out of 3) [18], Asn560Thr (1 out of 4) [23], and Thr556Ser (7 out of 9) [16, 18, 23, 29] have been previously reported in AMX-resistant and none of the susceptible *H. pylori* strains (Table 2). In our study, a binding site (Thr556Ser) mutation was only seen in 1 of the 4 resistant and none of the sequenced susceptible strains. In agreement with our findings, experimental induction of Thr556Ser mutation decreased the AMX susceptibility of the affected *H. pylori* strain, from 0.5 to 2 (mg/L) [16]. Similarly, the structural data on pneumococcal PBPs reveals that mutations surrounding the binding site impact the protein's total charge and polar character, leading to the encapsulation of the binding cleft [37]. A molecular dynamics simulation study of *Streptococcus pneumoniae* PBP1A showed that the key regions of the binding pocket in mutant strains were more flexible, allowing for the detachment of a third-generation β-lactam (cefotaxime) [38].

**Table 2:** Reported mutations in the PBP1A binding site and tunnel-1 residues of AMX-resistant and susceptible strains

Mutations	Binding site	Tunnel-1	No of strains R^1^/S^2^	Ref
Ala369Thr	✓	✓	3/4^R^–0/12^S^	[28]
Thr541Ilu	✓	–	1/3^R^–0/9^S^	[18]
Asn560Thr	✓	✓	1/4^R^–0/5^S^	[23]
Thr556Ser	✓	–	8/12^R^–0/19^S^	[16] [18] [23] [29] This study
Phe366Leu	–	✓	7/7^R^	[15]
Ser414Arg	✓	✓	31/104^R^–1/106^S^	[15] [18] [22] [28] [39] This study
Val469Met	–	✓	2/5^R^–0/11^S^	[24] This study

^1^Resistant

^2^Sensitive

Based on the crystal structure of *S. pneumoniae* PBP1A, mutations in the hotspot of the catalytic (binding) site entrance, could considerably change the tunnel entry characteristics by modifying surface polarity, which may, in turn, modify the drug accessibility of the mutated PBP1A binding site [25]. Accordingly, conformational mutations in tunnel-1 residues are expected to play a role in creating resistance, as they affect the drug’s access to the enzyme's active site. In our study, tunnel-1 (Ser414Arg, Val469Met) mutations were only seen in 2 of the 4 resistant and none of the 10 susceptible strains. In agreement with our findings, mutations in the tunnel-1 residues are also previously reported in AMX-resistant *H. pylori* strains (Table 2). These residues, in addition to Ala369Thr and Asn560Thr (stated above), include Phe366Leu (7 out of 7 resistant strains) [15], Ser414Arg (31 out of 104 resistant and only 1 out of 133 sensitive strains) [15, 18, 22, 28, 39], and Val469Met (2 out of 5 resistant and none of the 11 sensitive strains) [24]. The Ser414Arg mutation is the most frequently reported mutation in AMX-resistant *H. pylori* strains. Its determining role in AMX resistance is evidenced by increased MIC of the parent strain from 0.125 mg/L to 0.5–1 mg/L, in the experimentally mutated strain [15]. In agreement with previously published studies [28, 40], Ser414 is among the six critical sites (Ser414, Thr438, Phe473, Ser543, Thr556, and Asn562) for AMX binding to PBP1A. Three of these substitutions are previously reported in multiple clinical *H. pylori* strains (Table 2); Ser414Arg in tunnel-1, Thr556Ser in the binding site, and Asn562Tyr [24]. Taken together, these our findings on our limited number of clinical strains and those of others (Table 2), support the critical essence of the binding site and tunnel-1 residues, in potentially causing AMX resistance.

## Conclusions

To conclude, in the first step, using computational tools, we have identified the AMX binding site residues in PBP1A and the six tunnel-like routes accessing it. Accordingly, we and others have detected mutations in these amino acids, almost entirely in the AMX-resistant and not in the sensitive *H. pylori* strains. It can thus be assumed that these mutations may hinder AMX access to the catalytic Ser368 residue. Therefore, we hypothesize that conformational mutations in amino acid residues lining the binding site as well as tunnel-1, will likely cause AMX resistance, as they may block every route for AMX accessing and binding to PBP1A. More research, however, is required to accurately analyze the effects of these conformational changes, on drug binding, via crystallographic studies of the PBP1A in *H. pylori.*

## Materials and methods

### Computational methods

#### 3D structure prediction and tunnel detection in AMX binding to PBP1A

Due to lack of access to *H. pylori* PBP1A crystal structure, the I-TASSER server (https://zhanglab.ccmb.med.umich.edu/I-TASSER/) [41] was used to obtain a 3D structural model. The FASTA sequence of PBP1A for the reference (ATCC26695) strain was submitted as an input, without assigning any restraints or templates. The best-predicted model with the highest confidence was built from the most significant templates, in the threading alignments. This model had the closest structural similarity to that of *Staphylococcus aureus* PBP2 (PDB ID: 3DWK) on the Protein Data Bank (PDB) database (https://www.rcsb.org/). The model was minimized for 20,000 steps of the conjugate gradient method, with the CHARMM27 [42] force field in NAMD 2.13 [43] package. MolProbity [44] was used to validate the quality of the minimized structural model. To identify the tunnels of the PBP1A minimized structure, the CAVER 3.0 [45] software was used. The probe radius was set to 1 Å and the binding site was chosen as the starting point. Other CAVER parameters were set as default.

#### Covalent molecular docking of AMX with PBP1A

For molecular docking studies, the minimized conformation of the PBP1A and the AMX structure, which was obtained from the ZINC database (http://zinc.docking.org/), were used as the receptor and ligand, respectively. In order to attach the ligand to the receptor structure covalently, ligand alignment was performed. For ligand alignment, the receptor and ligand files, the ligand atom indices, and the SER368 catalytic residue were specified. The standard PDBQT files, the covalent ligand structures, rigid and flexible components PDBQT, AutoGrid, and AutoDock parameter files were prepared for docking, using MGLTools 1.5.6 [46]. Which also generated the rigid and flexible components PDBQT, AutoGrid, and AutoDock parameter files.

The docking box (with 27 × 28 × 30 Å dimensions) was defined around Ser368, as the catalytic residue for covalent interaction. The genetic algorithm was used as the searching algorithm with 200 runs. The “unbound_model bound” entry in the DPF file was manually edited to “unbound_energy 0.0”. All other parameters were set to default values. The AutoGrid and AutoDock 4.2 [47] programs were used according to standard procedures. The best covalent interaction of AMX-PBP1A, with the lowest free energy, was used for subsequent analysis.

The conformations were shown by VMD1.9.3 [48]. Finally, LigPlot^+^v.1.4 [49] analysis determined the PBP1A residues involved in interaction with AMX and their interaction types. Also, the COACH web server (https://zhanglab.ccmb.med.umich.edu/COACH/) [50] was used as a meta-server, to predict the protein–ligand binding site and compare the docking results.

### Experimental methods

#### Bacterial strains and growth conditions

One hundred clinical *H. pylori* isolates were collected from 290 dyspeptic patients, via upper endoscopy, from 2013 to 2018, at Amiralam Hospital, Tehran, Iran. Gastric biopsy specimens were cultured onto Brucella agar medium (Merck, Germany), supplemented with 10% defibrinated sheep blood, amphotericin B (8 mg/L), vancomycin (10 mg/L), and trimethoprim (5 mg/L) and incubated under microaerobic conditions (O2, 5%; CO2, 10%; N2, 85%) at 37 °C for 3–5 days [51]. Sample collection was performed according to the approved protocols by the Committee on Ethical Issues in Medical Research, Pasteur Institute of Iran (Ref.No.IR.PII.REC.1394.57) and every patient provided written informed consent.

#### Amoxicillin susceptibility testing

For each isolated *H. pylori* strain, a 3.0 McFarland standard bacterial suspension was prepared in 1 mL sterile saline. One hundred microliters of this bacterial suspension was spread onto Muller Hinton agar, with 7% (v/v) sheep blood, using sterile cotton swabs. E test (Epsilometer test, BioMerieux France) strips were placed onto the plates and incubated at 37 °C, under microaerobic conditions for three days [50]. Tested strains were considered resistant to AMX, if the minimum inhibitory concentrations (MIC) were: > 0.125 μg/mL [53].

#### Amplification of the *pbp1a* gene

The genomic DNA from *H. pylori* isolates were extracted and purified, using the DNA Micro Kit (Qiagen, USA). *Pbp1a* gene amplification was carried out by PCR, using primers PBP1-F TCGTTACAGACACGAGCACC) and PBP1-R (CGTGTTATCGTCCCTCCCAA) and Amp ONE™ αPfu DNA polymerase kit (GeneAll Biotechnology, South Korea). The primers were designed using Primer3 (NCBI), based on the *pbp1a* gene sequence of 26695 reference strain*.* The transpetidase domain of PBP1A, corresponding to nucleotides 998 to 1758 of *pbp1*a gene (> NC_000915.1) was amplified. The PCR reaction was carried out at 95 °C for 5 min, followed by 40 cycles of 95 °C for 10 s and 55 °C for 30 s, and final extension at 72 °C for 60 s. The expected PCR product was 761 bp. The *pbp1a* gene sequences, verified by Sanger sequencing at Pishgam Biotech Co., were deposited into the GenBank database, under the following accession numbers: MK984213-MK984221 & MK984223-MK984227. The obtained DNA sequences were aligned against that of ATCC 26695 reference strain. Sequence analysis was performed using the ClustalW sequence alignment tool, available in the CLC Main Workbench (version 5.5).

## Supplementary Information

**Additional file 1: File S1.** The top 10 threading templates for *H. pylori* PBP1A 3D structure modeling.

**Additional file 2: Figure S1.** Ramachandran plot of the minimized structure.

**Additional file 3: File S2.** The binding probability of PBP1A residues by COACH web server.

**Additional file 4: Figure S2.** Schematic view of mutations detected in the PBP1A binding site and tunnel-1 residues of AMX-resistant and sensitive strains.

## Data Availability

The gene sequences evaluated in the current study are available in the NCBI GenBank repository [https://www.ncbi.nlm.nih.gov/genbank/].

## Abbreviations

AMX: Amoxicillin
PBP: Penicillin binding protein
