# Supplementary material for: The penicillin binding protein 1A of Helicobacter pylori, its amoxicillin binding site and access routes
Source: Gut Pathog. 2021 Jun 28;13:43. doi: 10.1186/s13099-021-00438-0 (PMC8240269; doi:10.1186/s13099-021-00438-0)
Supplement: Supplementary file 2 — Additional file 2: Figure S1. Ramachandran plot of the minimized structure. [file 13099_2021_438_MOESM2_ESM.docx]

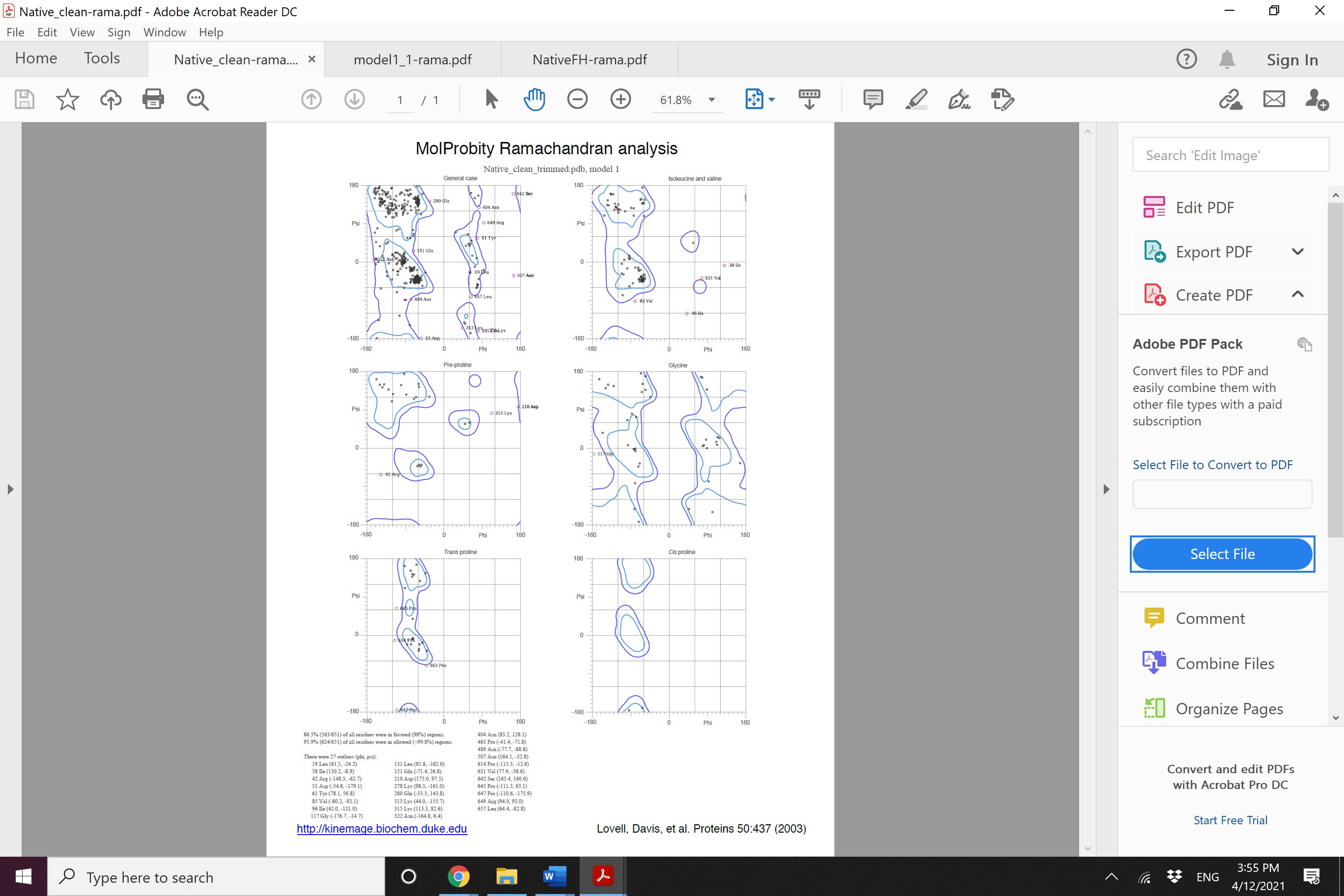


Figure S1. Ramachandran plot of the minimized structure. The most favorable regions and residues in the favored regions were shown in blue lines and dark gray points, respectively. Residues in less favorable regions were depicted in purple and red points.
