# Supplementary material for: The penicillin binding protein 1A of Helicobacter pylori, its amoxicillin binding site and access routes
Source: Gut Pathog. 2021 Jun 28;13:43. doi: 10.1186/s13099-021-00438-0 (PMC8240269; doi:10.1186/s13099-021-00438-0)
Supplement: Supplementary file 4 — Additional file 4: Figure S2. Schematic view of mutations detected in the PBP1A binding site and tunnel-1 residues of AMX-resistant and sensitive strains. [file 13099_2021_438_MOESM4_ESM.docx]

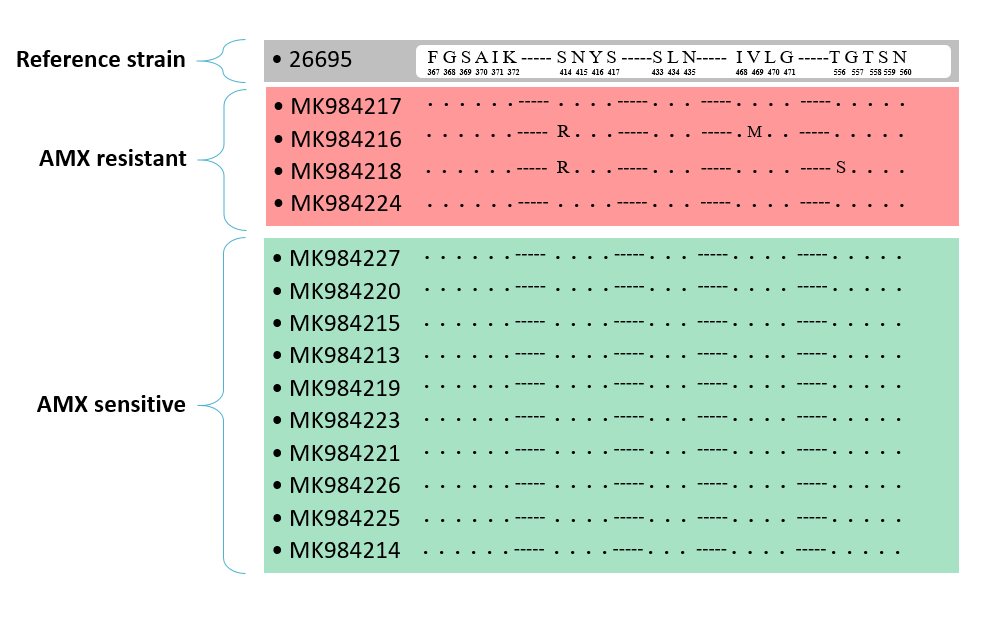


Figure S2. Schematic view of mutations detected in the PBP1A binding site and tunnel-1 residues of AMX-resistant and sensitive strains.
